# The effect of the COVID-19 pandemic on mental health in individuals with pre-existing mental illness

**DOI:** 10.1192/bjo.2022.25

**Published:** 2022-03-07

**Authors:** Katie J. S. Lewis, Catrin Lewis, Alice Roberts, Natalie A. Richards, Claudia Evison, Holly A. Pearce, Keith Lloyd, Alan Meudell, Bethan M. Edwards, Catherine A. Robinson, Rob Poole, Ann John, Jonathan I. Bisson, Ian Jones

**Affiliations:** National Centre for Mental Health, Division of Psychological Medicine and Clinical Neurosciences, Cardiff University, UK; National Centre for Mental Health, Division of Psychological Medicine and Clinical Neurosciences, Cardiff University, UK; National Centre for Mental Health, Division of Psychological Medicine and Clinical Neurosciences, Cardiff University, UK; National Centre for Mental Health, Division of Psychological Medicine and Clinical Neurosciences, Cardiff University, UK; National Centre for Mental Health, Division of Psychological Medicine and Clinical Neurosciences, Cardiff University, UK; National Centre for Mental Health, Division of Psychological Medicine and Clinical Neurosciences, Cardiff University, UK; Swansea Medical School, Swansea University, UK; Partnership in Research, National Centre for Mental Health, Division of Psychological Medicine and Clinical Neurosciences, Cardiff University, UK; Partnership in Research, National Centre for Mental Health, Division of Psychological Medicine and Clinical Neurosciences, Cardiff University, UK; Social Care and Society Research Group, School of Health Sciences, University of Manchester, UK; Centre for Mental Health and Society, Bangor University, UK; Population Data Science Group, Swansea University Medical School, Swansea University, UK; National Centre for Mental Health, Division of Psychological Medicine and Clinical Neurosciences, Cardiff University, UK; National Centre for Mental Health, Division of Psychological Medicine and Clinical Neurosciences, Cardiff University, UK

**Keywords:** COVID-19, depression, anxiety disorders, pre-existing mental illness, post-traumatic stress disorder

## Abstract

**Background:**

There is evidence that the COVID-19 pandemic has negatively affected mental health, but most studies have been conducted in the general population.

**Aims:**

To identify factors associated with mental health during the COVID-19 pandemic in individuals with pre-existing mental illness.

**Method:**

Participants (*N* = 2869, 78% women, ages 18–94 years) from a UK cohort (the National Centre for Mental Health) with a history of mental illness completed a cross-sectional online survey in June to August 2020. Mental health assessments were the GAD-7 (anxiety), PHQ-9 (depression) and WHO-5 (well-being) questionnaires, and a self-report question on whether their mental health had changed during the pandemic. Regressions examined associations between mental health outcomes and hypothesised risk factors. Secondary analyses examined associations between specific mental health diagnoses and mental health.

**Results:**

A total of 60% of participants reported that mental health had worsened during the pandemic. Younger age, difficulty accessing mental health services, low income, income affected by COVID-19, worry about COVID-19, reduced sleep and increased alcohol/drug use were associated with increased depression and anxiety symptoms and reduced well-being. Feeling socially supported by friends/family/services was associated with better mental health and well-being. Participants with a history of anxiety, depression, post-traumatic stress disorder or eating disorder were more likely to report that mental health had worsened during the pandemic than individuals without a history of these diagnoses.

**Conclusions:**

We identified factors associated with worse mental health during the COVID-19 pandemic in individuals with pre-existing mental illness, in addition to specific groups potentially at elevated risk of poor mental health during the pandemic.

The potential negative effect of the COVID-19 pandemic on mental health has been an area of concern.^1^ Research to date has found that symptoms of depression, anxiety and post-traumatic stress have increased during the pandemic.^2–4^ However, it is important to identify and examine populations who may be at greater risk of poorer mental health, including people with pre-existing mental health conditions. Recent studies suggest this population may be at increased risk of poor mental health during the COVID-19 pandemic compared with the general population,^5–8^ but findings are mixed.^9–12^

Potential explanations for adverse effects of the pandemic on people with pre-existing mental health problems include restricted access to mental health services^13^ and the psychological impact of social distancing.^14,15^ This was reflected in a survey of 2198 people, 70% of whom reported a history of mental illness, in March 2020. Key concerns about the COVID-19 pandemic included social isolation, access to mental health support/services and the financial impact of the pandemic.^1^ In addition, in the general population, groups who appear to be at greater risk of poor mental health during the pandemic include young people,^2–5,16^ women,^2,3,5,16^ being in an ethnic minority,^6^ caregivers^3,4,6^ and those at financial disadvantage and/or have lost employment because of the pandemic.^2,4,5^ Other factors previously associated with poor mental health during the pandemic include poor sleep^17^ and worry about COVID-19 infection.^6^ Finally, there have also been concerns about the effects of increased alcohol/drug use, particularly in those with existing drug use disorders.^18^ In terms of protective factors, there is evidence that mindfulness and social support buffer against negative COVID-19-related mental health outcomes.^19^

Further investigation of whether the COVID-19 pandemic has affected mental health in this population, and the factors associated with this, is needed. There is also a need to determine if the impact of the COVID-19 pandemic is the same across all mental health diagnoses, as different disorders have heterogeneous clinical characteristics, aetiologies and risk factors.^10^ It is imperative to examine whether the COVID-19 pandemic has differentially affected people with specific mental health diagnoses to inform interventions, ongoing care and policy.

## Aims

Using data from a large survey of people with pre-existing mental health conditions, we aimed to examine the impact of the pandemic on the mental health of people with pre-existing mental health diagnoses; and examine the associations of hypothesised factors with mental health outcomes (depression, anxiety and well-being). Based on existing research outlined above, we hypothesised that younger age, female gender, being in an ethnic minority group, having caring responsibilities, difficulty accessing mental health services, low income, income affected by COVID-19, worry about COVID-19, sleep loss and alcohol/drug use would be associated with worse mental health. Conversely, based on prior research finding that social isolation and loneliness is associated with poorer mental health,^5,6^ we hypothesised that feeling socially supported by friends, relatives or services would be associated with better mental health during the pandemic. Finally, we aimed to examine associations between specific mental health diagnoses and mental health during the COVID-19 pandemic. We did not have specific hypotheses about which diagnoses would be associated with worse mental health.

## Method

### Participants and study design

Participants were individuals with a history of mental illness or neurodevelopmental disorders recruited to the National Centre for Mental Health (NCMH), a Welsh Government-funded Research Centre that investigates neurodevelopmental and mental illness across the lifespan. The authors assert that all procedures contributing to this work comply with the ethical standards of the relevant national and institutional committees on human experimentation and with the Helsinki Declaration of 1975, as revised in 2008. All procedures involving human patients were approved by the Wales Research Ethics Committee (IRAS reference: 155838, REC reference: 16/WA/0323).

Participants were recruited systematically through primary and secondary healthcare services (via clinical care teams and screening of clinical notes) and non-systematically (e.g. advertising in local/national media, engaging third-sector organisations to promote the research). Due to the sampling strategy, the NCMH cohort is not a population cohort, but a targeted recruitment of participants with mental illness. It therefore includes a large proportion of participants who have had contact with mental health services and who have pre-existing mental illness. This includes, but is not limited to, neurodevelopmental disorders (e.g. attention-deficit hyperactivity disorder (ADHD)), depression, anxiety, schizophrenia, bipolar disorder, eating disorders, personality disorder and post-traumatic stress disorder (PTSD).

Participants joined the NCMH cohort from 2011 onward either taking part in a face-to-face interview with a researcher or completing an online assessment. Written informed consent was obtained from all participants. On joining the cohort, participants provided clinical information (including mental health diagnoses) and demographic data (e.g. gender, ethnicity, employment). Psychiatric diagnoses were derived by asking participants if they had ever received a psychiatric or neurodevelopmental diagnosis by a health professional.

### Online cross-sectional COVID-19 survey

In June 2020, participants aged 18 years or older who were enrolled in NCMH, had consented to contact for future research, had provided an email address and had a history of mental illness or neurodevelopmental disorders (*n* = 10 017/20 117), were invited to complete an online survey assessing the impact of the COVID-19 pandemic on their mental health. The survey included questions on demographic variables (current employment, current income, living arrangements) in addition to specific questions on the impact of the COVID-19 pandemic, shown in Table 1.

**Table 1:** Clinical and demographic information on the sample

Variable	*N* (%)
Age	1984 (69.2) ≥35 years
	873 (30.4) <35 years
	12 (0.4) missing
Gender	2227 (77.6) female
	3 (0.1) transgender female
	578 (20.1) male
	18 (0.6) transgender male
	39 (1.4) gender variant/non-conforming /non-binary
	4 (0.1) missing
Ethnicity	2725 (95.0) White
	113 (3.9) ethnic minority
	31 (1.1) missing
Employment	1491 (52.0) employed
	381 (13.3) retired
	261 (9.1) student
	730 (25.4) unemployed
	6 (0.2) missing
Approximate gross household income	675 (23.5) up to £10 000
	462 (16.1) £10 000–£20 000
	441 (15.4) £20 000–£30 000
	933 (32.5) over £30 000
	358 (12.5) missing
Highest level of qualification	73 (2.5) none/less than equivalent to GCSE
	548 (19.1) GCSE or equivalent
	558 (19.4) A level or equivalent
	1459 (50.9) Degree level or above
	231 (8.1) missing
Caring responsibilities	1720 (60.0) no
	865 (30.1) yes
	284 (9.9) missing
Keyworker	1983 (69.1) no
	815 (28.4) yes
	71 (2.5) missing
Work, study or employment status change	1317 (45.9) no
	1505 (52.5) yes
	47 (1.6) missing
Difficulty accessing mental health services	1649 (57.5) no
	697 (24.3) yes
	523 (18.2) missing

### Outcome measures

#### Anxiety

Anxiety over the preceding 2 weeks was assessed using the Generalised Anxiety Disorder seven-item questionnaire (GAD-7).^20^ This comprises seven questions, with scores ranging from 0 to 21. Higher scores indicate greater levels of anxiety, with scores ≥10 representing moderate levels of anxiety (89% sensitivity and 82% specificity).

#### Depression

Depression symptoms over the preceding 2 weeks were assessed using the nine-item Patient Health Questionnaire (PHQ-9).^21^ Scores range from 0 to 27, with higher scores indicating greater levels of depression symptoms. A PHQ-9 score of ≥10 is indicative of major depression (88% sensitivity and 85% specificity).^22^

#### Psychological well-being

Psychological well-being over the past 2 weeks was assessed using the World Health Organization five-item Well-Being Index (WHO-5).^23^ Scores range from 0 to 25, with higher scores indicating greater well-being. Scores <13 indicate poor well-being and are indicative of depression according to ICD-10 criteria. The WHO-5 has good construct validity as an indicator of well-being and as a screening tool for depression.^24^

#### Self-reported impact of the pandemic on mental health

Participants were asked, ‘During the COVID-19 crisis, how has your mental health been?’, with five possible responses: ‘Much better than usual’, ‘Better than usual’, ‘About the same as usual’, ‘Worse than usual’ and ‘Much worse than usual’. A binary variable was created with responses ‘Worse than usual/Much worse than usual’ (1) versus ‘Much better than usual/Better than usual/About the same as usual’ (0).

### Predictors

Based on literature from the general population and the data available in our survey, we chose the following predictors: age (analysed as a mean-centred continuous variable); gender (women versus men); being in an ethnic minority group; caring responsibilities; difficulty accessing mental health services; low income; financial impact of the COVID-19 pandemic; social support by friends, relatives and services; worries about COVID-19; reduced sleep and increased alcohol/drug use.^2,3,5,6,16–18,25–27^ Further information on the COVID-19 survey question wording and coding of responses is provided in Supplementary Table 1 available at https://doi.org/10.1192/bjo.2022.25.

#### Diagnostic groups

Participants selected all mental health and neurodevelopmental diagnoses they had received from a list (options provided in the Supplementary Material). Diagnoses were grouped as follows: anxiety, depression, obsessive–compulsive disorder (OCD), bipolar disorder, schizophrenia/psychosis, PTSD, eating disorder, personality disorder, alcohol/other drug misuse, autism spectrum disorders (ASD) and ADHD. Participants were included in multiple diagnostic groups apart from those with bipolar disorder and schizophrenia/psychosis, who were excluded from the anxiety and depression groups. We also created a ‘number of comorbidities’ variable, indicating how many mental health diagnoses each participant had received.

### Analysis

We performed linear regressions of each predictor on each of the mental health outcomes. Sensitivity analyses adjusted for multiple testing with the Holm method, and adjusted for potential confounders (age, gender and income). Regression analyses assume that data are missing at random, which could lead to bias if this assumption is violated. Potential bias due to missing data was addressed in Stata (version 15.1 for Mac), using the multivariable imputation by chained equations algorithm.^28^ The imputation models included all variables included in the primary analyses, in addition to auxiliary variables. Auxiliary variables were those collected in the survey or at study entry that were found to be predictive of missingness and were associated with scores of the variables to be imputed (e.g. similar measures collected at other points in the survey or at other assessments). These included information on education, employment status, worry about mental health and the age at onset of psychiatric symptoms. One hundred imputed data-sets were generated for analysis, with estimates combined using Rubin's rules.^29^ This procedure increased the plausibility of the missing at random assumption. Details on the models used for imputation of each outcome variable are provided in the Supplementary Material.

## Results

### Participant characteristics

Out of 10 017 invited individuals, 3137 participants completed the survey, but one participant later withdrew from the study, resulting in 3136 (31%) participants (mean age 45 years, range 18–94 years). Using variables collected at enrolment into the NCMH cohort, which had complete or near-complete data, we examined which variables were associated with non-response to the survey invitation. We found that participants who were recruited via the National Health Service, were of younger age, were in an ethnic minority group, male and who had never been employed were less likely to respond to the survey invitation. In addition, participants with ADHD, schizophrenia, psychosis, bipolar disorder, ASD and a history of alcohol or drug misuse were less likely to respond to the survey. Full results are shown in Supplementary Table 2.

Before analysis, we excluded participants who reported that they had not received or did not disclose a diagnosis of a mental health/neurodevelopmental condition (*n* = 267). This resulted in a final sample of 2869 eligible individuals for the primary analyses. Table 1 summarises the demographic information of the sample. The majority of participants (70.1%) completed the survey in the week commencing 15 June 2020, with the rest of the sample completing the survey between 26 June 2020 and 30 July 2020.

### Predictors of poor current mental health

**Fig. 1:**
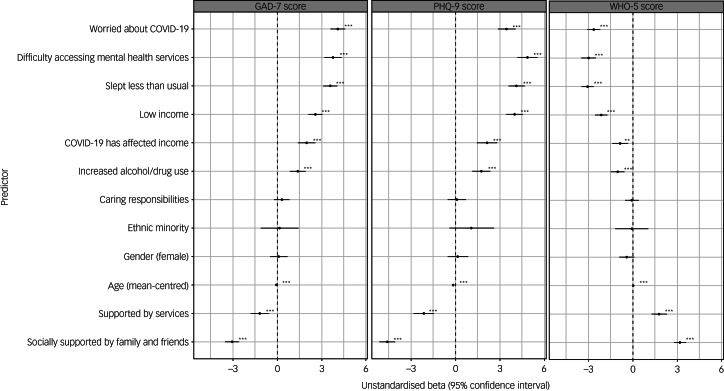
Results of linear regressions showing associations between hypothesised predictors with GAD-7, PHQ-9 and WHO-5 total scores during the COVID-19 pandemic. All predictors are binary except for age, which was mean-centred. Estimates shown after correction for confounders. Asterisks shown for estimates that survived correction for multiple testing and adjustment for potential confounders (*P < 0.05, **P < 0.01, ***P < 0.001). Error bars indicate 95% confidence intervals for the estimate. GAD-7, Generalised Anxiety Disorder seven-item scale; PHQ-9, Patient Health Questionnaire nine-item scale; WHO-5, World Health Organization five-item Well-Being Index.

**Fig. 2:**
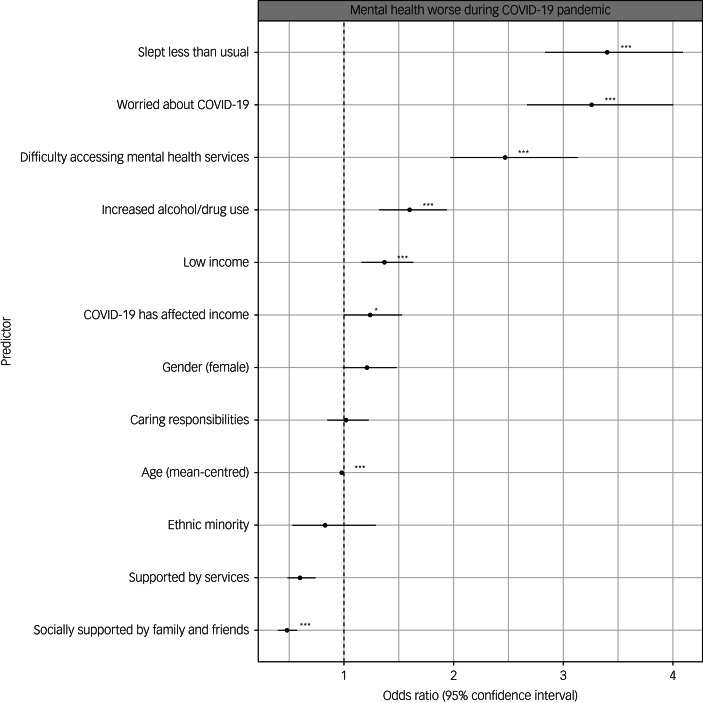
Results of logistic regressions showing associations between hypothesised predictors and whether participants reported that their mental health had worsened during the COVID-19 pandemic. All predictors are binary except for age, which was mean-centred. Estimates shown after correction for confounders. Asterisks shown for estimates that survived correction for multiple testing and adjustment for confounders (*P < 0.05, **P < 0.01, ***P < 0.001). Error bars indicate 95% confidence intervals for the estimate.

Within the sample, average (s.d.) scores on the GAD-7, PHQ-9 and WHO-5 were 10.07 (6.16), 12.76 (7.30) and 8.53 (5.24), respectively. The percentage of participants who were in the clinical range for each questionnaire (≥10 for the GAD-7 and PHQ-9, <13 for the WHO-5) were 46% (GAD-7), 58% (PHQ-9) and 73% (WHO-5). When asked about their mental health during the pandemic, 60% reported that their mental health had worsened, 10% reported that it had got better and 28% reported that it had stayed the same (3% had missing data for this variable). Fig. 1 and Fig. 2 summarise the regression analyses results after adjusting for confounders (all results shown in Supplementary Tables 3 and 4). Variables associated with poorer current mental health (depression, anxiety and reporting that mental health had worsened during the pandemic) and lower well-being after adjusting for multiple testing and adjusting for confounders (age, gender and income) were younger age, reporting difficulty accessing mental health services, low income, income affected by the COVID-19 pandemic, worrying about COVID-19, sleeping less than usual and drinking alcohol/taking drugs more than usual. Key predictors with large effect sizes across all mental health outcomes are highlighted below.

Being worried about the COVID-19 pandemic was associated with a 4.09-point increase (95% CI 3.60–4.48) in GAD-7 score, a 3.50-point increase (95% CI 2.92–4.09) in PHQ-9 score and a 2.63-point decrease (95% CI −3.07 to −2.20) in WHO-5 score. These participants had 3.26 (95% CI 2.67–4.00) increased odds of reporting that their mental health had worsened during the pandemic.

Difficulty accessing mental health services was associated with a 3.77-point increase (95% CI 3.20–4.34) in GAD-7 score, a 4.92-point increase (95% CI 4.25–5.59) in PHQ-9 score and 2.98-point reduction (95% CI −3.48 to −2.49) in WHO-5 score. People who had difficulty accessing mental health services had 2.47 (95% CI 1.97–3.12) increased odds of reporting that their mental health had worsened during the pandemic.

Reporting sleeping less than usual was associated with a 3.58-point increase (95% CI 3.11–4.05) in GAD-7 score, a 4.17-point increase (95% CI 3.63–4.71) in PHQ-9 score and a 3.05-point reduction (95% CI –3.45 to –2.65) in WHO-5 score. Participants who slept less than usual had 3.40 (95% CI 2.84–4.08) increased odds of reporting that their mental health had worsened during the pandemic.

Factors associated with better mental health and well-being were older age, feeling socially supported by family and/or friends, and feeling supported by services.

### Historical diagnoses associated with measures of current mental health

**Fig. 3:**
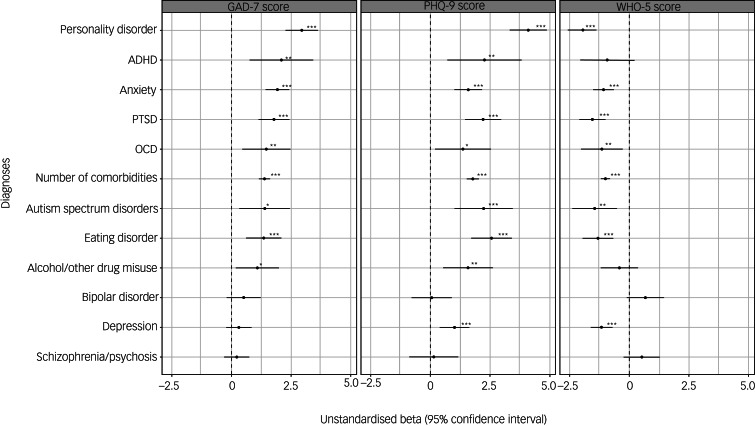
Results of linear regressions showing associations between psychiatric and neurodevelopmental diagnoses with GAD-7, PHQ-9 and WHO-5 total scores during the COVID-19 pandemic. All predictors are binary except for age, which was mean-centred. Estimates shown after correction for confounders. Asterisks shown for estimates that survived correction for multiple testing and adjustment for potential confounders (*P < 0.05, **P < 0.01, ***P < 0.001). Error bars indicate 95% confidence intervals for the estimate. ADHD, attention-deficit hyperactivity disorder; GAD-7, Generalised Anxiety Disorder seven-item scale; OCD, obsessive–compulsive disorder; PHQ-9, Patient Health Questionnaire nine-item scale; PTSD, post-traumatic stress disorder; WHO-5, World Health Organization five-item Well-Being Index.

As shown in Fig. 3, after adjusting for multiple testing and confounders, we found that participants with a history of personality disorder, ADHD, anxiety, PTSD, ASD, eating disorder or alcohol/other drug misuse had higher GAD-7 scores compared with those without these diagnoses. These same diagnoses were also associated with higher PHQ-9 scores and lower WHO-5 scores, with the exception of ADHD and alcohol/other drug misuse, which were not associated with lower WHO-5 scores after adjusting for confounders. Finally, a history of depression was associated with higher PHQ-9 and lower WHO-5 scores. The largest effect sizes were observed for those with a history of personality disorders.

**Fig. 4:**
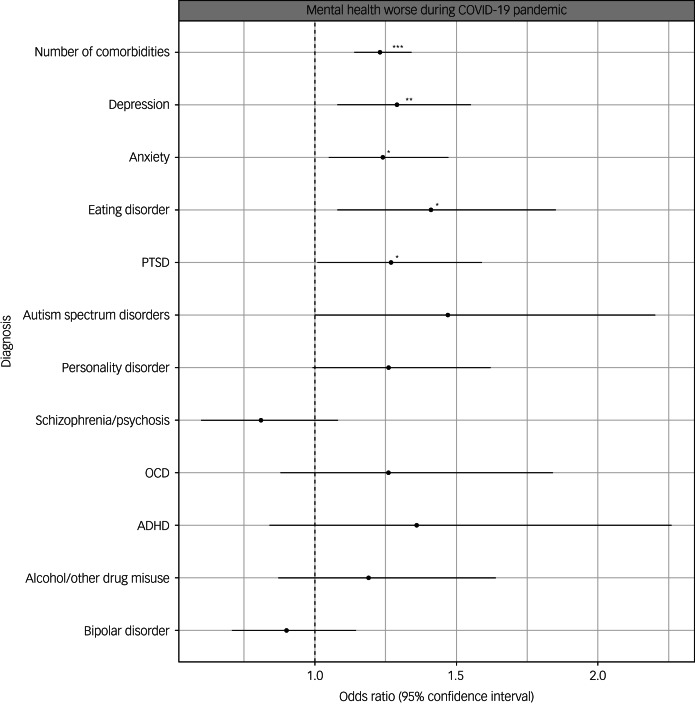
Results of logistic regressions showing associations between psychiatric and neurodevelopmental diagnoses and whether participants reported that their mental health had worsened during the COVID-19 pandemic. All predictors are binary except for age, which was mean-centred. Estimates shown after correction for confounders. Asterisks shown for estimates that survived correction for multiple testing and adjustment for potential confounders (*P < 0.05, **P < 0.01, ***P < 0.001). Error bars indicate 95% confidence intervals for the estimate. ADHD, attention-deficit hyperactivity disorder; OCD, obsessive–compulsive disorder; PTSD, post-traumatic stress disorder.

After sensitivity analyses correcting for multiple testing and adjusting for confounders, we found that participants with a history of anxiety, depression, PTSD and eating disorders were more likely to report that their mental health had worsened during the COVID-19 pandemic than individuals with other diagnoses (Fig. 4). Having more than one mental health condition (number of comorbidities) was associated with worse mental health across all measures.

### Results using imputed data

Complete data on predictors, confounders and outcome variables for each analysis ranged from 1957 to 2792 out of 2869 participants. The pattern of results for analyses using the imputed data (and controlling for confounders) were similar to those from the main analyses (see Supplementary Tables 5 and 6). Exceptions to this were results for gender, ADHD and ASD. In the imputed results, female gender was associated with self-report that mental health had worsened during the pandemic (odds ratio 1.22, 95% CI 1.01–1.48, *P* = 0.040). ASD was also associated with self-report that mental health had worsened during the pandemic (odds ratio 1.50, 95% CI 1.04–2.13, *P* = 0.028). Finally, ADHD was associated with lower WHO-5 scores (*B* = −1.13, 95% CI −2.19 to −0.10, *P* = 0.032).

## Discussion

In our study of people with pre-existing mental health conditions, we observed high levels of anxiety and depression symptoms and low levels of well-being during the pandemic. The mean levels of anxiety and depression symptoms, as measured by the GAD-7 (10.07) and PHQ-9 (12.76), exceeded those observed in studies of the UK population conducted in March to April 2020 (GAD-7 = 5.7–8.0, PHQ-9 = 6.6–9.0^30–32^). The mean score of the WHO-5 in our study (8.53) was also lower than that observed in a study of the UK population in April 2020 (13.0^31^). A large proportion of our sample scored in the clinical range of the GAD-7 (46%), PHQ-9 (58%) and WHO-5 (73%), indicating moderate levels of anxiety and depression, and low well-being. The proportion scoring in the clinical range for the GAD-7 and PHQ-9 (i.e. scores ≥10) was higher than that reported in studies of the UK general population from March to May 2020 (GAD-7:  17–39%, PHQ-9: 24–41%^4,7,30,31^), and higher than the rates reported in a UK general population sample over the same period our survey was conducted (GAD-7: 11.5–14.5%, PHQ-9: 16.3–21.0%^32^).

It is possible that the levels of poor mental health observed in our study do not reflect a change from pre-pandemic levels. However, we were able to assess whether participants thought that their mental health had changed since the pandemic began. Sixty per cent of participants in our study thought their mental health had worsened during the pandemic. This is in line with existing research findings in the UK general population,^7,30,31^ and highlights that those with pre-existing mental illness are likely to be particularly vulnerable to worsened mental health during the pandemic. We are currently collecting longitudinal data in this cohort to further explore the impact of the pandemic in this population.

When examining specific factors, we found that younger age, difficulty accessing mental health services, low income, financial impact of the COVID-19 pandemic, worry about COVID-19, sleeping less than usual and increased alcohol/other drug intake were associated with higher levels of anxiety, depression and poor well-being. We observed the same pattern of results when the outcome was participants’ perception of whether their mental health had worsened during the pandemic. Our results provide additional evidence that these factors are associated with worse mental health during the pandemic for people with pre-existing mental illness. This is consistent with results from previous research in the general population.^4–6,16,31,32^ The results for younger participants are particularly important, given that concerns have already been raised about the effect of the COVID-19 pandemic on young people and how pre-existing psychopathology may amplify these detrimental effects.^33^

In contrast to research in the general population,^6,30,31,34,35^ we did not find that female gender, caregiving responsibilities or being from an ethnic minority background were associated with worse mental health. These could reflect interactions with socioeconomic adversity^36,37^ or pre-existing mental illness. When using imputed data that minimised potential bias due to missing data, we also found that women were more likely to report that their mental health had worsened during the pandemic, but did not differ from men on other measures of mental health. This highlights the complex relationship between gender and mental health during the pandemic and future research should explore the mechanisms of this perhaps including a wider range of mental health measures. It should also be noted that 95% of our sample was White, and minority ethnic individuals in the NCMH cohort were less likely to complete the survey. In addition, dichotomising ethnicity into ethnic minority versus White might obscure associations with specific ethnicities. Due to small numbers, we were unable to examine the impact of gender beyond a self-report male versus female dichotomy, an important issue as one study found that non-binary people were at risk of worse mental health during the pandemic.^6^ Future research including more diverse samples is warranted.

Feeling supported by family and friends and by services were associated with better mental health. This is in line with existing research on the psychological impact of quarantine^14^ and the association between social isolation and mental health in those with lived-experience of mental disorders.^38^ A recent study of people with schizophrenia, schizoaffective disorder, bipolar disorder and major depressive disorder with psychotic features found that increased well-being during the COVID-19 pandemic was associated with spending less time alone pre-pandemic.^10^ However, given the cross-sectional nature of our study, it is possible that people with worse mental health are more likely to report feeling less supported or are in greater need of the support of clinical services.

In secondary analyses, we examined associations between specific mental health diagnoses and mental health during the COVID-19 pandemic. People with pre-existing anxiety, OCD, PTSD, eating disorders, ASD and personality disorders had worse scores across all measures of current mental health (GAD-7, PHQ-9 and WHO-5). The association between GAD-7 scores and many of these disorders, including anxiety disorders, OCD and PTSD, could be expected, given that anxiety symptoms are core to these diagnoses, although this study assessed lifetime and not current diagnoses. Other diagnoses were associated only with some measures of current mental health; participants with a history of depression (perhaps unsurprisingly) displayed higher levels of current depressive symptoms and lower well-being, but not current anxiety, whereas participants with ADHD had higher levels of current anxiety and depression, but not lower well-being after controlling for confounders. However, ADHD was associated with lower well-being when using imputed data, suggesting that the non-significant result for well-being may have been the result of bias from missing data. Of note, the effect sizes for a history of eating disorders and personality disorders were particularly large. For example, having a history of a personality disorder was associated with an over four-point increase in PHQ-9 scores after controlling for confounders. These results illustrate that having more than one pre-existing mental health diagnosis was associated with increased anxiety and depression scores, reduced well-being and being more likely to report that mental health had deteriorated during the pandemic. This is consistent with another COVID-19 study that found worsening mental health with increasing mental health comorbidities in a Dutch population.^9^

When examining whether participants thought their mental health had changed during the pandemic, we found that people with pre-existing anxiety, depression, PTSD and eating disorders were more likely to report that mental health had worsened. In addition, when using imputed data, we also found that this was associated with a diagnosis of ASD. This suggests these groups could be at high risk of deteriorated mental health during the pandemic. The fact that the diagnoses associated with this outcome were not the same as those associated with current mental health symptoms suggests that assessments of current mental health might not reflect increases relative to pre-pandemic levels for all groups. However, longitudinal assessments are necessary to verify this.

We did not find that bipolar disorder or schizophrenia/psychosis were associated with worse current mental health scores compared with other disorders. However, we did not assess specific mental health symptoms associated with these conditions and, as for all participants, it is possible that people struggling the most would be less likely to respond to the request to participate. Another study conducted during the pandemic also found individuals diagnosed with schizophrenia, schizoaffective disorder, bipolar disorder and major depressive disorder with psychotic features displayed stable mental health compared with pre-pandemic measures.^10^ The authors posit that these results could reflect regression to the mean or reflect the benefits of increased structure and fixed routines as a consequence of social distancing measures.^10^ Additional possibilities are that restrictions imposed by lockdowns might reduce social demands, which, in turn, could improve mental health in some people. However, although it is possible that people with bipolar disorder or schizophrenia/psychosis might have greater resilience to the effects of the pandemic (for example, more experience of dealing with social isolation), responses to effects of the pandemic could also have been influenced by medication. For example, some research has shown that, compared with people with anxiety and depression, individuals with schizophrenia experience lower post-traumatic stress symptoms in response to major traumatic events.^39^ Further research needs to explore the mechanisms underpinning these findings. More longitudinal research is needed to investigate if this pattern of results will be sustained as the impact of the pandemic continues.

### Strengths and limitations

Strengths of our study include the use of data on a large sample of individuals with a history of mental health conditions using validated standardised measures. We examined the impact of the COVID-19 pandemic on individuals who had received a range of psychiatric diagnoses, which allowed us to draw comparisons between specific groups. Furthermore, we addressed potential bias resulting from missing data by using multiple imputation, in which we were able to draw on auxiliary information from other areas of the survey and prior assessments to minimise potential bias.

Our study has several limitations. First, data were cross-sectional, therefore we cannot determine the direction of effect between predictor and outcome variables. This is particularly important when assessing whether mental health changed during the pandemic, as in the current study we were only able to examine participants’ perceived change in mental health. However, we are collecting follow-up data that will allow for the identification of factors that prospectively predict worse mental health. Second, the diagnoses recorded included all current and past diagnoses that participants had received. We therefore did not know whether participants were experiencing an episode of illness at the time they completed the survey. We also did not know what treatment (if any) participants were currently receiving, which could have influenced results. Third, our diagnoses were self-report with, for the majority of participants, no in-depth diagnostic interview, clinician report or data from medical records. However, preliminary work from our group has found a positive predictive value of 85% when comparing a self-reported diagnosis of schizophrenia or schizoaffective disorder (depressed episode) to research diagnosis, using an in-depth psychiatric interview.^40^

Fourth, 50% of our sample were educated to degree level or above and 95% were White. It should be noted, however, that a large proportion of the sample resided within Wales, which is less ethnically diverse than England, with 93% reportedly White in the 2011 census.^41^ Compared with the NCMH sample as a whole, we found that participants who did not complete the survey differed from the NCMH cohort in a number of ways, including being younger, more likely to be male and from an ethnic minority group. We also found that participants in the NCMH cohort with ADHD, schizophrenia/psychosis, ASD and bipolar disorder were less likely to complete the survey (Supplementary Table 2). Our sample may therefore have lacked representativeness of individuals with mental health problems and of participants in the NCMH cohort as a whole. It will be important for future research in this area to identify how best to engage these groups and improve retention. Emerging research suggests that planned retention protocols that strengthen participant–researcher relationships through robust participant-tracking systems and participant engagement activities (e.g. establishing a rapport, regular follow-up interviews) could be effective strategies.^42^ We also identified that number of years since recruitment was associated with increased odds of not responding to the survey (Supplementary Table 2), suggesting that efforts to engage more longstanding members of the NCMH cohort will be particularly important.

### Future work

There are a number of areas that future work should focus on. One area that we did not explore in this study was factors associated with positive mental health outcomes. For example, 10% of participants in our study reported that their mental health had improved during the pandemic. We are currently conducting qualitative studies with these participants to identify which factors may have contributed to this.

In addition, we used brief measures that assessed mental health symptoms over the prior 2 weeks (PHQ-9 and GAD-7). These are widely used measures and allowed us to compare our results with those from studies of the general population during the pandemic. However, future longitudinal research is needed that uses mental health measures that can delineate what aspects, for example, of anxiety were most affected by the pandemic (e.g. post-traumatic stress symptoms), and how these change throughout the pandemic.

Finally, although we focussed on symptoms of depression, anxiety and on general well-being, there are other aspects of mental health we did not obtain data on that may be important. For example, psychotic symptoms, symptoms of high mood episodes, eating disorder and OCD behaviours would not have been captured in our study.

### Clinical and policy implications

Our results have a number of implications for both research and clinical practice. First, although scores of current mental health symptoms were high and well-being low compared with reports in the general population, there were large individual differences in people with pre-existing mental health conditions. In fact, 10% of our participants reported that their mental health improved during the pandemic. It is important, therefore, not to assume that all those with a history of mental illness will do badly. In addition, our results suggest that there are differences in the impact of the pandemic across diagnoses, with certain conditions (anxiety, depression, PTSD and eating disorders) at high risk of worsened mental health.

Second, our study identified several factors that were associated with low well-being and poor current mental health. In particular, we found that participants reporting difficulty accessing mental health services and feeling unsupported by services had poor current mental health. These findings highlight the importance of ensuring continued access to services for individuals with mental health conditions, and corroborate calls to prioritise this aspect of care.^6^

Third, those participants at greatest socioeconomic disadvantage (low income and being financially affected by the pandemic) were found to have worse current mental health. This is of particular importance, given the ongoing and predicted long-term economic impact of the pandemic. This finding suggests that addressing socioeconomic disadvantage should be a target for prevention strategies, and will be an important area for policy makers to address in addition to the provision of mental health services.

In conclusion, the present study identified that people with pre-existing mental health problems have experienced high levels of depression and anxiety symptoms and lower well-being during the COVID-19 pandemic. A large proportion of participants reported that their mental health had deteriorated during the pandemic, particularly those with a history of anxiety, depression, PTSD or eating disorders. The greatest impact was reported by participants who felt poorly supported, who had difficulties accessing services and those at socioeconomic disadvantage. Further research on the longitudinal associations between mental health and the COVID-19 pandemic in those with pre-existing mental health disorders is needed to understand these associations.

## Data Availability

The data that support the findings of this study are available from the corresponding author, I.J., upon reasonable request.

## Supplementary material

For supplementary material accompanying this paper visit https://doi.org/10.1192/bjo.2022.25. click here to view supplementary material
