# Supplementary material for: The effect of the COVID-19 pandemic on mental health in individuals with pre-existing mental illness
Source: BJPsych Open. 2022 Mar 7;8(2):e59. doi: 10.1192/bjo.2022.25 (PMC8914135; doi:10.1192/bjo.2022.25)
Supplement: Supplementary file 1 [file S2056472422000254sup001.docx]

# Supplementary Material

**Table S1.** Question wording, responses and coding used in the COVID-19 survey.

| Variable | Survey Question | Response coding |
| --- | --- | --- |
| Caring responsibilities | Do you have caring responsibilities? | (0) I do not have caring responsibilities(1) I have caring responsibilities for children under the age of 18/I have caring responsibilities for an adult with a disability/ I have caring responsibilities for an elderly person |
| Difficulty accessing mental health services | Have you had difficulty getting access to mental health services during the COVID-19 crisis? | (0) No (1) Yes |
| Low income | What is your current approximate annual household income before tax? | (1) Only benefits/Up to £10,000/£10,000- £20,000(0) £20,000- £30,000 £30,000- £40,000 £40,000- £50,000 £50,000- £60,000 ≥ £60,000 |
| Financial impact of the COVID-19 pandemic | As a result of COVID-19, have you experienced… | (1) Significant loss of income/Been unable to pay bills/ Been unable to afford sufficient food/Needed to apply for emergency loans or hardship funds/Needed to access a food bank. (0) Did not endorse any of the above |
| Felt socially supported by family and friends | In the last two weeks, how socially supported by family/friends have you felt? | (0) Very poorly/Poorly/Neither poorly nor well/Not applicable (1) Well/Very well |
| Felt socially supported by services | In the last two weeks, how socially supported by services have you felt? | (0) Very poorly/Poorly/Neither poorly nor well/ (1) Well/Very well |
| Worried about COVID-19 | In the last two weeks, how worried have you been about COVID-19? | (0) Not at all worried/ A little worried/Somewhat worried(1) Worried/Very worried |
| Slept less than usual | Has COVID-19 had an effect on your activities? Over the last two weeks have you done these things more, about the same, or less than before the COVID-19 crisis. – Got enough sleep | (0) About the same as usual/more than usual/much more than usual(1) Much less than usual/less than usual |
| Increased alcohol/drug use | Has COVID-19 had an effect on your activities? Over the last two weeks have you done these things more, about the same, or less than before the COVID-19 crisis. Drunk alcohol  Smoked cannabis  Used other recreational drugs | (0) About the same as usual/less than usual/ much less than usual(1) More than usual/much more than usual |

# Diagnoses

For the question, “What mental health or neurodevelopmental diagnosis or condition have you been given or received treatment for?”, participants could select the following options: depressive disorder (depression, major depression), bipolar disorder (manic depression), mania (hypomania), schizophrenia, schizoaffective disorder, psychosis, anxiety (generalised anxiety disorder, GAD), agoraphobia, panic disorder, phobias, obsessive compulsive disorder (OCD), autism, Asperger’s syndrome, autism spectrum disorder, anorexia, bulimia, binge eating disorder, borderline personality disorder (emotionally unstable personality disorder), other personality disorder, alcohol dependence/misuse, dependence/misuse of other drugs, mood disorder in pregnancy, postpartum psychosis (postnatal psychosis / puerperal psychosis), postnatal depression (postpartum depression), attention deficit hyperactivity disorder (ADHD), post-traumatic stress disorder (PTSD), complex post-traumatic stress disorder (CPTSD), and dementia. Participants were also able to indicate if they had received a diagnosis of any other psychiatric disorder not listed. Participants could select multiple diagnoses.

# Non-Response to the COVID-19 Survey Invitation

**Table S2.** Variables associated with non-response to the COVID-19 survey invitation (univariate regressions).

| **Variable** | **OR** | **95% CI** | ***p*** |
| --- | --- | --- | --- |
| Recruited through NCMH field team or NHS | 1.91 | 1.75,2.09 | <.001 |
| ADHD | 1.58 | 1.22,2.06 | .001 |
| Ethnic minority | 1.50 | 1.22,1.86 | <.001 |
| Severe Mental Illness (Schizophrenia/Psychosis/Bipolar Disorder) | 1.38 | 1.24,1.54 | <.001 |
| Alcohol or Drug Misuse | 1.37 | 1.17,1.62 | <.001 |
| Autism Spectrum Disorder | 1.32 | 1.09,1.62 | .006 |
| Number of years since recruited | 1.13 | 1.10,1.16 | <.001 |
| Number of comorbidities | 1.06 | 1.03,1.09 | <.001 |
| Age | 0.99 | 0.98,0.99 | <.001 |
| Anxiety | 0.84 | 0.78,0.92 | <.001 |
| Depression | 0.77 | 0.71,0.84 | <.001 |
| Ever employed | 0.72 | 0.65,0.79 | <.001 |
| Gender (female) | 0.71 | 0.64,0.79 | <.001 |
| Recruited online | 0.47 | 0.43,0.51 | <.001 |

ADHD, Attention-Deficit Hyperactivity Disorder; BAME, Black, Asian and Minority Ethnicity.

# Multiple Imputation

Depending on the analysis, 1949 to 2405 out of 2869 participants had complete data on predictors, confounders and outcome variables. We used multiple imputation by chained equations (Royston & White, 2011) to impute GAD-7, PHQ-9 and WHO-5 scores. These models used auxiliary information collected from other parts of the COVID-19 survey and from prior NCMH assessments. We selected auxiliary variables for imputation models based on theory, their association with values in the outcome variables, and their association with missingness. We did not include variables that were only associated with missingness in outcome variables. In addition to auxiliary variables associated with observed data and missingness, imputation models included all variables in the main analyses (including the outcome) and sensitivity analyses. These steps made the missing at random assumption more plausible. Each imputation model generated 100 imputed datasets. We used the two-stage quadratic rule (von Hippel, 2020) to check that the number of imputed datasets was sufficient to ensure replicable standard error estimates.

In the imputation model with GAD-7 score as the outcome, the following variables were included (in addition to those in the main analyses): PHQ-9 score, WHO-5 score, mental health worsened, education, employment status, number of comorbidities, age at onset of symptoms, worry about the effect of COVID-19 on mental health, worry about the effect of COVID-19 on physical health, worry about the effect of COVID-19 on others, worry about the effect of COVID-19 on financial situation, displayed symptoms of COVID-19, needed to self-isolate, living in a household with less than the median number of rooms, socialised face to face much less than usual.

In the imputation model with PHQ-9 score as the outcome, the following variables were included (in addition to those in the main analyses): GAD-7 score, WHO-5 score, mental health worsened, education, employment status, number of comorbidities, age at onset of symptoms, worry about the effect of COVID-19 on mental health, suicidal thoughts as a consequence of the COVID-19 pandemic, self-harm as a consequence of the COVID-19 pandemic, used self-management techniques to support mental health less, received less support from family and friends for mental health, relationships with family/friends got worse, used self-care for mental health less, access to mental health services changed, socialised remotely less, difficulty accessing medication, had to follow shielding advice.

In the imputation model with WHO-5 scores as the outcome, the following variables were included (in addition to those in the main analyses): GAD-7 score, PHQ-9 score, mental health worsened, education, employment status, number of comorbidities, age at onset of symptoms, worry about the effect of COVID-19 on mental health, diagnosis of severe mental illness, spent less time on relaxing activities.

In the imputation model with mental health worsened as the outcome, the following variables were included (in addition to those in the main analyses): GAD-7 score, WHO-5 score, PHQ-9 score, education, employment status, number of comorbidities, age at onset of symptoms, worry about the effect of COVID-19 on mental health, spent less time on relaxing activities, relationships with family/friends got worse, socialised face to face much less, suicidal thoughts as a consequence of the COVID-19 pandemic.

**Table S3.** Variables associated with mental health outcomes during the COVID-19 pandemic.

*Adjusted for age, gender and income. Results which surpassed sensitivity analyses for both (1) correction for multiple testing and (2) adjustment for confounders shown in bold.

**Table S4.** Diagnoses associated with mental health outcomes during the COVID-19 pandemic.

*Adjusted for age, gender and income. Results which surpassed sensitivity analyses for both (1) correction for multiple testing and (2) adjustment for confounders shown in bold. ADHD, Attention-Deficit Hyperactivity Disorder; OCD, Obsessive-compulsive disorder; PTSD, post-traumatic stress disorder; CPTSD, complex post-traumatic stress disorder.

**Table S5**. Variables associated with mental health outcomes during the COVID-19 pandemic (using imputed data).

|  |  | **Imputed data (adjusted for confounders*)** | | |
| --- | --- | --- | --- | --- |
| **Outcome** | **Predictor** | **B** | **95% CI** | ***p*** |
| GAD-7 | Age | -0.10 | -0.11,-0.08 | **<.001** |
|  | Gender (female) | -0.05 | -0.60,0.50 | .853 |
|  | Ethnic minority | -0.20 | -1.35,0.96 | .738 |
|  | Caring responsibilities (yes) | 0.28 | -0.20,0.77 | .251 |
|  | Difficulty accessing mental health services | 3.99 | 3.46,4.52 | **<.001** |
|  | Low income (income <£20,000) | 2.39 | 1.92,2.85 | **<.001** |
|  | COVID-19 has impacted income | 1.91 | 1.37,2.45 | **<.001** |
|  | Socially supported by family and friends | -2.76 | -3.19,-2.33 | **<.001** |
|  | Supported by services | -1.13 | -1.68,-0.58 | **<.001** |
|  | Worried about COVID-19 | 4.20 | 3.75,4.64 | **<.001** |
|  | Slept less than usual | 3.62 | 3.20,4.05 | **<.001** |
|  | Increased alcohol/drug use | 1.36 | 0.87,1.85 | **<.001** |
| PHQ-9 | Age | -0.12 | -0.14,-0.10 | **<.001** |
|  | Gender | 0.02 | -0.62,0.66 | .945 |
|  | Ethnic minority | 0.92 | -0.43,2.27 | .181 |
|  | Caring responsibilities (yes) | 0.09 | -0.48,0.66 | .764 |
|  | Difficulty accessing mental health services | 5.18 | 4.57,5.79 | **<.001** |
|  | Low income (income <£20,000) | 3.79 | 3.24,4.34 | **<.001** |
|  | COVID-19 has impacted income | 2.29 | 1.67,2.92 | **<.001** |
|  | Socially supported by family and friends | -4.29 | -4.78,-3.80 | **<.001** |
|  | Supported by services | -1.98 | -2.62,-1.34 | **<.001** |
|  | Worried about COVID-19 | 3.56 | 3.03,4.09 | **<.001** |
|  | Slept less than usual | 4.31 | 3.82,4.81 | **<.001** |
|  | Increased alcohol/drug use | 1.54 | 0.96,2.11 | **<.001** |
| WHO-5 | Age | 0.05 | 0.04,0.06 | **<.001** |
|  | Gender (female) | -0.32 | -0.80,0.15 | .183 |
|  | Ethnic minority | -0.09 | -1.09,0.91 | .866 |
|  | Caring responsibilities (yes) | -0.13 | -0.55,0.29 | .544 |
|  | Difficulty accessing mental health services | -3.04 | -3.50,-2.59 | **<.001** |
|  | Low income (income <£20,000) | -1.99 | -2.40,-1.58 | **<.001** |
|  | COVID-19 has impacted income | -0.90 | -1.37,-0.43 | **<.001** |
|  | Socially supported by family and friends | 3.13 | 2.76,3.49 | **<.001** |
|  | Supported by services | 1.68 | 1.21,2.16 | **<.001** |
|  | Worried about COVID-19 | -2.55 | -2.94,-2.15 | **<.001** |
|  | Slept less than usual | -3.06 | -3.43,-2.69 | **<.001** |
|  | Increased alcohol/drug use | -0.89 | -1.32,-0.46 | **<.001** |
| **Outcome** | **Predictor** | **OR** | **95% CI** | ***p*** |
| Mental health worse during the COVID-19 pandemic | Age | 0.98 | 0.98,0.99 | **<.001** |
|  | Gender (female) | 1.22 | 1.01,1.48 | **.040** |
|  | Ethnic minority | 0.89 | 0.59,1.33 | .563 |
|  | Caring responsibilities (yes) | 1.02 | 0.86,1.20 | .849 |
|  | Difficulty accessing mental health services | 2.48 | 2.00,3.07 | **<.001** |
|  | Low income (income <£20,000) | 1.35 | 1.14,1.59 | **<.001** |
|  | COVID-19 has impacted income | 1.27 | 1.05,1.55 | **.016** |
|  | Socially supported by family and friends | 0.51 | 0.43,0.59 | **<.001** |
|  | Supported by services | 0.66 | 0.54,0.79 | **<.001** |
|  | Worried about COVID-19 | 3.28 | 2.72,3.94 | **<.001** |
|  | Slept less than usual | 3.53 | 2.98,4.18 | **<.001** |
|  | Increased alcohol/drug use | 1.58 | 1.32,1.88 | **<.001** |

Results are adjusted for age, gender and income.

**Table S6**. Diagnoses associated with mental health outcomes during the COVID-19 pandemic (using imputed data).

|  |  | **Imputed data (adjusted for confounders*)** | | |
| --- | --- | --- | --- | --- |
| **Outcome** | **Predictor** | **B** | **95% CI** | ***p*** |
| GAD-7 | Anxiety | 1.90 | 1.44,2.34 | **<.001** |
|  | OCD | 1.44 | 0.52,2.37 | **.002** |
|  | Depression | 0.37 | -0.12,0.84 | .141 |
|  | Bipolar Disorder | 0.48 | -0.16,1.17 | .137 |
|  | Schizophrenia/Psychosis | 0.28 | -0.54,1.07 | .519 |
|  | PTSD | 2.01 | 1.42,2.58 | **<.001** |
|  | Eating Disorder | 1.49 | 0.80,2.14 | **<.001** |
|  | Personality Disorder | 2.81 | 2.15,3.41 | **<.001** |
|  | Alcohol/other drug misuse | 1.31 | 0.48,2.18 | **.002** |
|  | Autism Spectrum Disorders | 1.49 | 0.55,2.43 | **.002** |
|  | ADHD | 2.11 | 0.91,3.34 | **.001** |
|  | Number of comorbidities | 1.41 | 1.23,1.61 | **<.001** |
| PHQ-9 | Anxiety | 1.58 | 1.06,2.10 | **<.001** |
|  | OCD | 1.32 | 0.23,2.38 | **.017** |
|  | Depression | 1.05 | 0.49,1.60 | **<.001** |
|  | Bipolar Disorder | 0.29 | -0.45,1.08 | .420 |
|  | Schizophrenia/Psychosis | 0.11 | -0.85,1.01 | .869 |
|  | PTSD | 2.46 | 1.78,3.13 | **<.001** |
|  | Eating Disorder | 2.64 | 1.83,3.38 | **<.001** |
|  | Personality Disorder | 3.78 | 3.01,4.46 | **<.001** |
|  | Alcohol/other drug misuse | 1.74 | 0.76,2.71 | **<.001** |
|  | Autism Spectrum Disorders | 2.33 | 1.23,3.42 | **<.001** |
|  | ADHD | 2.49 | 1.11,3.92 | **<.001** |
|  | Number of comorbidities | 1.77 | 1.56,2.00 | **<.001** |
| WHO-5 | Anxiety | -1.06 | -1.45,-0.67 | **<.001** |
|  | OCD | -1.05 | -1.86,-0.26 | **0.009** |
|  | Depression | -1.15 | -1.55,-0.72 | **<.001** |
|  | Bipolar Disorder | 0.52 | -0.07,1.07 | .086 |
|  | Schizophrenia/Psychosis | 0.51 | -0.18,1.22 | .144 |
|  | PTSD | -1.61 | -2.11,-1.11 | **<.001** |
|  | Eating Disorder | -1.36 | -1.93,-0.76 | **<.001** |
|  | Personality Disorder | -1.95 | -2.48,-1.38 | **<.001** |
|  | Alcohol/other drug misuse | -0.59 | -1.31,0.15 | .117 |
|  | Autism Spectrum Disorders | -1.45 | -2.24,-0.61 | **0.001** |
|  | ADHD | -1.13 | -2.19,-0.10 | **.032** |
|  | Number of comorbidities | -1.01 | -1.18,-0.85 | **<.001** |
| **Outcome** |  | **OR** | **95% CI** | ***p*** |
| Mental health worse during the COVID-19 pandemic | Anxiety | 1.21 | 1.04,1.43 | **.014** |
|  | OCD | 1.07 | 0.77,1.50 | .657 |
|  | Depression | 1.25 | 1.06,1.48 | **.007** |
|  | Bipolar Disorder | 0.98 | 0.78,1.24 | .906 |
|  | Schizophrenia/Psychosis | 0.80 | 0.60,1.04 | .096 |
|  | PTSD | 1.29 | 1.05,1.60 | **.016** |
|  | Eating Disorder | 1.46 | 1.14,1.89 | **.003** |
|  | Personality Disorder | 1.21 | 0.96,1.53 | .101 |
|  | Alcohol/other drug misuse | 1.20 | 0.89,1.62 | .239 |
|  | Autism Spectrum Disorders | 1.50 | 1.04,2.13 | **.028** |
|  | ADHD | 1.21 | 0.78,1.90 | .388 |
|  | Number of comorbidities | 1.22 | 1.14,1.32 | **<.001** |

*Adjusted for age, gender and income. ADHD, Attention-Deficit Hyperactivity Disorder; OCD, Obsessive-compulsive disorder; PTSD, post-traumatic stress disorder.
